# GPT2: a glucose 6-phosphate/phosphate translocator with a novel role in the regulation of sugar signalling during seedling development

**DOI:** 10.1093/aob/mct298

**Published:** 2014-01-31

**Authors:** Beth C. Dyson, Rachel E. Webster, Giles N. Johnson

**Affiliations:** 1 Faculty of Life Sciences, University of Manchester, Manchester M13 9PT, UK; 2 The Manchester Museum, University of Manchester, Oxford Road, Manchester M13 9PL, UK

**Keywords:** Germination, greening, *Arabidopsis thaliana*, glucose, sugar signalling, GPT2, seedling development

## Abstract

**Background and Aims:**

GPT2, a glucose 6-phosphate/phosphate translocator, plays an important role in environmental sensing in mature leaves of *Arabidopsis thaliana*. Its expression has also been detected in arabidopsis seeds and seedlings. In order to examine the role of this protein early in development, germination and seedling growth were studied.

**Methods:**

Germination, greening and establishment of seedlings were monitored in both wild-type *Arabidopsis thaliana* and in a *gpt2* T-DNA insertion knockout line. Seeds were sown on agar plates in the presence or absence of glucose and abscisic acid. Relative expression of *GPT2* in seedlings was measured using quantitative PCR.

**Key Results:**

Plants lacking *GPT2* expression were delayed (25–40 %) in seedling establishment, specifically in the process of cotyledon greening (rather than germination). This phenotype could not be rescued by glucose in the growth medium, with greening being *hyper*sensitive to glucose. Germination itself was, however, *hypo*sensitive to glucose in the *gpt2* mutant.

**Conclusions:**

The expression of *GPT2* modulates seedling development and plays a crucial role in determining the response of seedlings to exogenous sugars during their establishment. This allows us to conclude that endogenous sugar signals function in controlling germination and the transition from heterotrophic to autotrophic growth, and that the partitioning of glucose 6-phosphate, or related metabolites, between the cytosol and the plastid modulates these developmental responses.

## INTRODUCTION

The regulation of germination and early seedling development is complex and is controlled in response to a number of factors, including nutrient status, phytohormones, water availability and metabolite levels (Bewley, 1997). The timing of germination and subsequent seedling development is crucial to ensuring the success of the seedling (Bewley, 1997; Koornneef *et al.*, 2002). Seeds must develop fully before dispersal, and mature seeds must avoid germination within the maternal silique or in unfavourable conditions after dispersal. Fully mature, dry arabidopsis seeds are capable of surviving long periods of adverse conditions in a state of dormancy (Bentsink *et al.*, 2010), in order to establish seedlings in the best possible environmental conditions (Donohue, 2002). Dormancy is a property of a developed, viable seed, defined as an internal seed condition that blocks germination despite environmental conditions being sufficient for the process to occur (Bewley, 1997; Baskin and Baskin, 2004). In *Arabidopsis thaliana*, the major form of dormancy is referred to as primary dormancy, the level of which is dependent on the environment in which the seeds mature and are dispersed as well as the environmental conditions after dispersal (Koornneef *et al.*, 2002).

The transition from dependence on seed reserves to autotrophic growth is reliant on the mobilization of accumulated carbon and nutrient stores within the cotyledons (Graham, 2008; Kelly *et al.*, 2011). In arabidopsis seeds, carbon is stored mainly in the form of triacylglycerols, with starch and storage proteins forming a smaller proportion of the reserves necessary for seedling development (Graham, 2008). After germination these large, insoluble storage molecules need to be converted into smaller metabolites, including sucrose, which can be transported to the developing organs. Mutants impaired in the ability to mobilize metabolites can exhibit severely retarded germination and seedling development (Kelly *et al.*, 2011) or can fail to develop without exogenous metabolite application (Hayashi *et al.*, 1998; Baker *et al.*, 2006), showing that seed reserves are critical for the proper growth of the emerging seedling.

The transition to autotrophy requires the presence of fully developed chloroplasts and is marked by greening of the cotyledons. In arabidopsis this greening process requires light-induced production of chlorophyll and the differentiation of proplastids into true chloroplasts (Kim and Apel, 2004). Chloroplast development in cotyledons begins during embryogenesis, but is suspended during the process of seed maturation, beginning again after germination. If germination occurs under the soil surface, plastids will develop into etioplasts, using energy reserves from the cotyledons, until exposure to light stimulates the production of chlorophyll and development of true chloroplasts. When seedlings develop on agar, chloroplast development begins from proplastids immediately following germination, as cotyledons are immediately exposed to light and light-dependent chlorophyll production can begin. Chloroplast differentiation in cotyledons is a distinct pathway from the biogenesis of chloroplasts in true leaves, as shown by the snowy cotyledon (*sco*) mutant, which has pale cotyledons but has normal true leaf development (Albrecht *et al.*, 2006). Chloroplasts in cotyledons are similar to young leaf chloroplasts, containing less extensive thylakoid membranes than mature leaf chloroplasts (Deng and Gruissem, 1987). Successful seedling development therefore requires the germination of seeds, the mobilization of storage reserves, the production of chloroplasts in the cotyledons and the production of true leaves. These processes are tightly regulated to ensure seedlings emerge and develop under optimum environmental conditions.

Many important developmental processes, including germination and seedling growth, are regulated in response to metabolite levels, particularly to fluctuations of carbohydrates within the cell (Smith and Stitt, 2007; Smeekens, 2000; Rolland *et al.*, 2006). To maintain the balance between the production of carbohydrates and their use, plants need to have sensing and signalling mechanisms to effect suitable responses to any changes in carbohydrate levels (Gupta and Kaur, 2005; Corbesier *et al.*, 1998). Sugars have been shown to have signalling roles in a number of plant processes, including germination and seedling development, as well as growth, and regulation of photosynthesis, carbon metabolism and senescence. A number of these sugar signalling pathways and their key components have been identified in arabidopsis. For example, studies using mutants with altered responses to sugar have highlighted a signalling pathway involving the enzyme hexokinase as a sugar sensor (Jang *et al.*, 1997; Price *et al.*, 2003). Hexokinase catalyses the first step in glycolysis, the phosphorylation of glucose to glucose 6-phosphate, but has also been identified as having a sugar-sensing role that is independent of its catalytic function (Xiao *et al.*, 2000). Seeds and seedlings lacking hexokinase activity are insensitive to glucose in their growth medium, whilst over-expression lines are hypersensitive to low levels of exogenous glucose (Jang *et al.*, 1997).

A number of genes have been identified as being up-regulated by exogenous increases in sugar, including At1g61800, encoding a glucose 6-phosphate transporter, GPT2 (Knappe *et al.*, 2003). GPT2 is involved in the transport of glucose 6-phosphate across plastid membranes in return for inorganic phosphate (Niewiadomski *et al.*, 2005). Microarray analyses have shown that *GPT2* expression has been associated with impaired carbon metabolism (Kunz *et al.*, 2010), senescence (Pourtau *et al.*, 2006) and increases in carbon fixation due to increased light (Athanasiou *et al.*, 2010). In the latter study, it was demonstrated that expression of *GPT2* is required for plants to sense or respond normally to the increases in light that lead to acclimation of photosynthesis. This implies that it plays a role in sugar sensing, either directly (as a sensor itself) or, more likely, indirectly by affecting the balance of metabolites between cellular compartments.

*GPT2* expression has been reported in imbibed seeds and developing seedlings (Ruuska *et al.*, 2002; Finch-Savage *et al.*, 2007). *GPT2* has also previously been shown to be induced by the exogenous application of sugars to seedlings (Gonzali *et al.*, 2006). Sugars are known to influence both germination and seedling development. In light of this, we here examine the roles of GPT2 during germination and seedling development.

We show that plants lacking *GPT2* expression have slower early seedling growth, which can be attributed to the slower onset of cotyledon greening. In addition, germination of *gpt2* seeds is insensitive to moderate levels of glucose, whilst the greening delay is exacerbated by glucose in the growth medium. We show therefore that a lack of *GPT2* expression has a significant impact on seedling development, and modulates the sensing of sugar status during early seedling development.

## MATERIALS AND METHODS

### Plant material

Seeds of *Arabidopsis thaliana* ecotypes ‘Wassilewskija-2’ (Ws) and ‘Columbia 0’ (Col 0) were used in these experiments, along with their respective knockouts of the *GPT2* gene. A T-DNA insertion line in a Ws-2 background was obtained from the INRA Versailles collection (FLAG_326E03) and a homozygous insertion line (*gpt2-2*) was isolated (Athanasiou *et al.*, 2010). A homozygous T-DNA insertion line in a Col-0 background (*gpt2-1*) was isolated from GABI-KAT line GK-454H06-018837 (Li *et al.*, 2003). Seeds used in germination, greening and establishment assays were from plants grown under controlled environmental conditions on soil at 100 μmol m^2^ s^−1^ light, 8 h day/16 h night, 20 °C/18 °C until flowering. Seed assays were carried out within 2 months of seed harvest.

### Seedling development assays

All assays were performed on 1·5 % w/v agar containing half-strength Murashige and Skoog (MS) medium. PEG-8000 was used as an osmotic control. The indicated concentrations of glucose were added to the agar before pouring plates. PEG-8000 plates were made up according to van der Weele *et al.* (2000) to give the correct osmotic potential for comparison with 4 % w/v glucose, as agar containing 4 % PEG-8000 will not set. These plates were prepared as follows: 20 ml 1·5 % w/v agar containing half-strength MS medium was allowed to solidify in a Petri dish before the addition of 30 ml filter sterilized half-strength MS medium containing 275 g l^−l^ PEG-8000. Plates were allowed to soak for 18 h before excess liquid was poured away. This induced a water potential equivalent to 4 % glucose (approximately –0·55 MPa). Before sowing, seeds were surface-sterilized with 10 % v/v bleach, washed with distilled, sterile water, rinsed with 70 % v/v ethanol and then washed a further five times with distilled, sterile water. After 3 days of stratification at 4 °C in the dark, plates were transferred to 100 μmol m^2^ s^−1^ light with 8 h day/16 h night. Germination, defined as emergence of the radicle, was scored every 12 h. Greening and establishment were scored at the same time intervals. Each plate contained 50–150 seeds and experiments were performed in triplicate. Percentage germination and/or greening was calculated daily and plotted against time, and sigmoidal curves using a growth-based function were fitted in Origin (OriginLab, MA, USA) to determine the time at which 50 % of the seedlings had reached a particular developmental stage (T_50_). One-way or two-way analysis of variance calculations were performed in SPSS (IBM, UK).

### Seedling length data

Seeds were sown and stratified as above and allowed to grow for 3 days in upright Petri dishes. Seedling lengths were measured after three full photoperiods by photographing the plates under a microscope and analysing with ImageJ (National Institutes of Health, USA). Significance was determined using two-way ANOVA with a *post hoc* test, Tukey's B test (SPSS).

### Gene expression data

Seeds were sterilized and sown as for seedling development assays, on plates containing 1·5 % w/v agar with half-strength MS, 4 % w/v glucose or the osmotic equivalent of 4 % w/v PEG-8000. Nylon mesh was placed below the seeds on the plates to enable removal of the seeds and seedlings without contaminating samples with agar. Seeds/seedlings were removed from plates at the end of each photoperiod from the third to the seventh photoperiod after transfer to light. Samples were flash-frozen in liquid nitrogen, then stored at –80 °C until extraction of RNA. Total RNA was extracted from seed samples using the phenol:chloroform method as described by Oñate-Sánchez and Vicente-Carbajosa (2008) (their method 2). These RNA samples were treated with DNase to remove any contaminating DNA using the RQ1 enzyme (Promega, UK). One microgram of this RNA sample was used in a cDNA synthesis reaction using an MMLV reverse transcription enzyme (Bioline, UK). Quantitative PCR (qPCR) was carried out using primers for *GPT2*, *ACT2* and *UBC* as described in Athanasiou *et al.* (2010). qPCR was carried out in a one-step reaction using a Roche SYBRgreen Master Mix (with ROX) in an Applied Biosystems ABI Prism 7000 instrument. Data analysis was performed using the software ABI Prism 7000 sequence detection system version 1·7 (Applied Biosystems). Three RNA samples from each time point were assayed in triplicate, and expression levels were compared with the levels of two housekeeping genes, *ACT2* and *UBC*. Analysis of Ct values to produce relative expression data was performed according to Livak and Schmittgen (2001).

## RESULTS

### Cotyledon establishment is delayed in *gpt2* plants

To examine the effect of *GPT2* expression early in development, arabidopsis ecotypes Ws-2 and Col 0, as well as their respective T-DNA insertion mutants of the *GPT2* gene (*gpt2-2* and *gpt2-1*, respectively), were grown on agar medium containing half-strength MS. After a 2-day dark stratification at 4 °C and subsequent transfer to light (20 °C), establishment of the cotyledons was scored every 12 h. Cotyledon establishment was defined as the presence of green, fully open cotyledons (cotyledons having an angle of at least 180° between them).

**Fig. 1.**
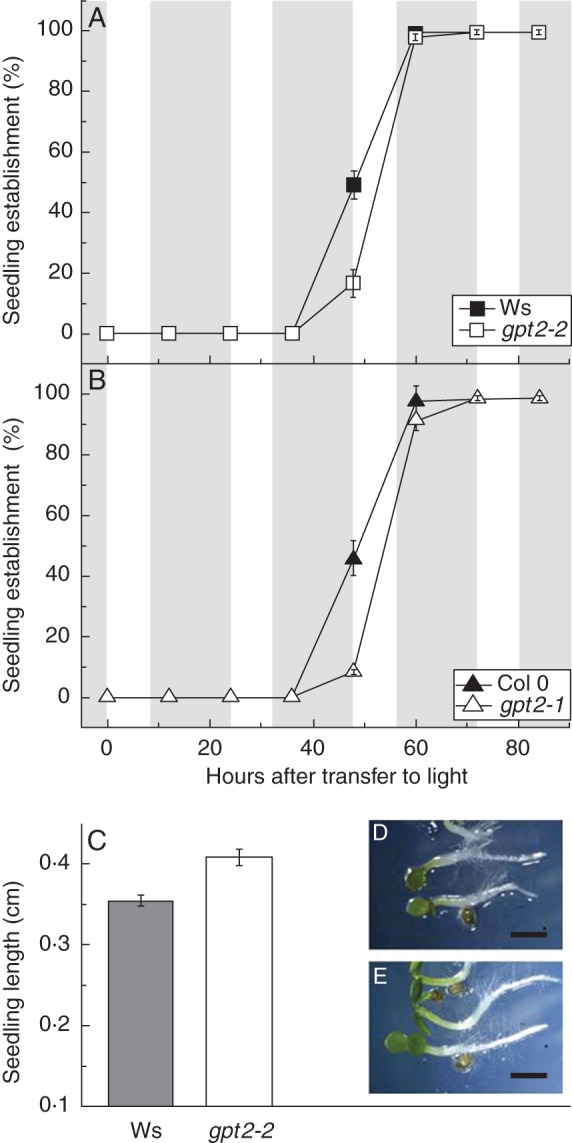
Seedling establishment in wild-type and *gpt2* plants. Seeds of wild-type *Arabidopsis thaliana* Ws-2 and Col 0 and their respective T-DNA insertion knockouts of *GPT2* (*gpt2-2* and *gpt2-1*) were sown on half-strength MS, 1·5 % agar plates after surface sterilization with 10 % bleach. After a 3-day stratification in the dark at 4 °C, plates were moved to 100 μmol m^2^ s^−1^ light on an 8-h photoperiod (shaded areas represent dark periods and unshaded areas represent photoperiods). (A, B) Seedling establishment was scored every 12 h as the presence of fully open green cotyledons. Between 50 and 150 seeds were sown on each plate, and each data point represents the mean of three to five plates. Error bars represent ±1 s.e. (C) Seedling lengths were measured in Ws-2 wild-type and *gpt2-2* plants after three full photoperiods in the light by photographing the plates under a dissection microscope and analysing with ImageJ (National Institutes of Health, USA). Error bars represent ±1 s.e. Significance was determined using two-way ANOVA with a *post hoc* test, Tukey's B test (SPSS). (D) Representative wild-type seedlings. (E) Representative *gpt2* seedlings. Scale bars = 8 mm.

Cotyledon establishment was significantly slowed in plants of both lines lacking *GPT2* expression (Fig. 1). The *gpt2* plants developed fully green, fully open cotyledons on average 13–15 h later than wild-type plants. Development of the first pair of true leaves in plants of both lines lacking *GPT2* occurred 12–15 h later than in wild-type (data not shown), indicating that there were no additional delays in seedling establishment. This delay led to a visible difference in the size of *gpt2* seedlings over the first days of post-germinative growth; however, rates of establishment approached 100 % in both wild-type and *gpt2* plants (Fig. 1A). Seedling growth is a function of both shoot and root growth; therefore, to examine overall plant growth during establishment, hypocotyl length was measured at the end of the third photoperiod (56 h) post-stratification (Fig. 1B–D) in wild-type Ws-2 and *gpt2-2* plants. At this point during seedling development, the majority of seedlings had fully established cotyledons (Fig. 1A). Despite establishment occurring later, *gpt2-2* plants were significantly longer (around 15 %, *P* < 0·04) than wild-type Ws-2 plants.

### The timing of germination is similar in wild-type and knockout plants, but greening is delayed in plants lacking GPT2

**Fig. 2.**
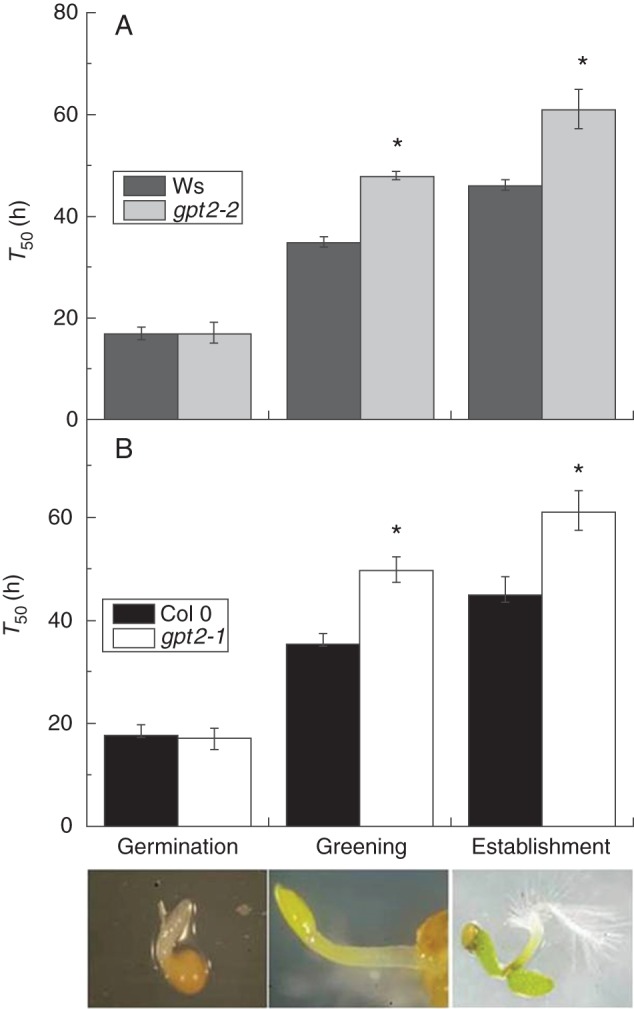
Mean time taken for 50 % (T_50_) of seeds/seedlings to achieve germination, greening and establishment (illustrated at bottom) in wild-type and *gpt2* plants on MS. Seeds of Ws-2, Col 0, *gpt2-2* and *gpt2-1* lines were sown, stratified and transferred to light as for seedling development assays. Germination was scored as the emergence of the radicle from the seed coat, greening as the presence of green cotyledons and establishment as the presence of fully open green cotyledons. Seeds of Ws-2 and *gpt2-2* (A) and Col 0 and *gpt2-1* (B) were examined every 12 h, and percentages of germination, greening and establishment were calculated for each plate on the basis of the number of seeds sown. These data were graphed and sigmoidal curves based on growth parameters were fitted (see Supplementary Data Figs 1 and 2). The mean time taken for 50 % (T_50_) of seeds/seedlings to achieve germination, greening and establishment was extrapolated from these graphs. Each data point is the mean of three to five replicate plates. Pictures show a representative example of the relevant developmental stage. Error bars represent ±1 s.e. ANOVAs were carried out separately on germination, greening and establishment data, and asterisks show significant differences (*P* ≤ 0·05) between lines at a particular developmental stage.

There are a number of processes occurring between imbibition and the establishment of open cotyledons, including germination, mobilization of seed reserves, chloroplast biogenesis and chlorophyll production. In order to better define processes that were delayed in *gpt2* plants, the mean time taken from imbibition and transfer to light for 50 % of seeds/seedlings (T_50_) to germinate (defined as emergence of the radicle), green (the presence of green cotyledons) or become established (as before) were examined (Fig. 2). In arabidopsis, cotyledon greening and opening take place concurrently. However, during environmental perturbations or stress, cotyledons may green and stay closed, or open without greening. These two processes are therefore examined separately here.

The timing of germination was unaltered in plants lacking *GPT2* expression; however, greening and subsequent establishment were significantly slowed in the two mutant lines (Fig. 2). The mean time taken for 50 % of seeds to germinate (T_50_ germination) was on average 16–18 h post-stratification in all wild-type and *gpt2* lines (for fitted curves see Supplementary Data Figs 1 and 2). The mean time taken for 50 % of seeds to green (T_50_ green) was 15–19 h later in both wild-type lines, with establishment complete shortly afterwards. Plants lacking *GPT2* expression exhibited a delay in greening, such that there was a gap of 30–32 h between germination and greening in these two mutant lines. Again, establishment occurred very soon after greening. The delay between germination and greening was therefore responsible for the slow development phenotype seen in the *gpt2* lines.

To further investigate this delayed greening and the potential explanations for it, the responses of the *gpt2* lines to glucose were investigated. Glucose is known to rescue certain slow growth phenotypes in arabidopsis, and the application of exogenous sugars is known to affect the growth of seedlings (Jang *et al.*, 1997). We postulated that, as a sugar transporter, GPT2 may have an effect on reserve mobilization between germination and greening. To examine the effects of glucose on cotyledon development in the absence of *GPT2*, greening was examined in wild-type and *gpt2* plants on MS containing varying concentrations of glucose.

### Germination is delayed in wild-type plants and greening is delayed in both wild-type and knockout plants under high concentrations of exogenous glucose

**Fig. 3.**
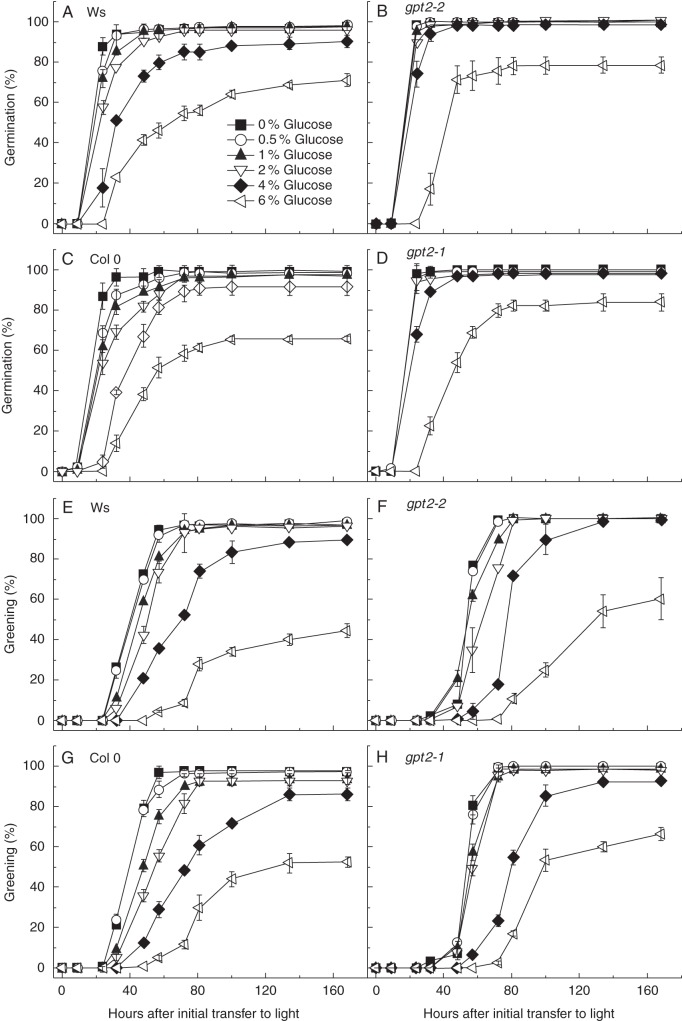
Germination and greening profiles of wild-type and *gpt2* seedlings on different concentrations of glucose. Seeds of Ws-2, Col 0, *gpt2-2* and *gpt2-1* lines were sterilized and sown on plates containing half-strength MS, 1·5 % agar and various concentrations of glucose. Seeds were scored every 12 h for germination or the presence of green cotyledons. Percentage greening was calculated for each plate on the basis of the number of seeds sown and each data point is the mean of three to five replicate plates. Error bars represent ±1 s.e. (A, E) Germination and greening in Ws-2 wild-type plants. (B, F) Germination and greening in *gpt2-2* plants. (C, G) Germination and greening in Col 0 wild-type plants. (D, H) Germination and greening in *gpt2-1* plants.

The responses of wild-type *Arabidopsis* seedlings to glucose have been well documented. Seedlings grown on media containing high levels of glucose fail to mobilize their seed reserves (To *et al.*, 2002), accumulate anthocyanins (Mita *et al.*, 1997; Solfanelli *et al.*, 2006) and may even fail to develop chloroplasts (To *et al.*, 2003). At the low concentrations of glucose (0·5–2 %), the number of seedlings germinating or developing green cotyledons was not affected; however, the rate of development was slowed as the glucose concentration increased (Fig. 3). Both *gpt2-1* and *gpt2-2* lines responded similarly to increasing levels of glucose in the growth media. At higher glucose concentrations (4–6 % w/v), wild-type plants did not develop normally; germination was delayed and seedling development was impaired (Fig. 3). Seed viability in all wild-type and *gpt2* lines approached 100 % (Fig. 1); any reduction in the number of seeds that germinated is therefore likely to be due to increased dormancy. Inclusion of 6 % glucose in the growth medium resulted in an approximately 30 % reduction in the number of seeds that germinated over the 160 h experimental period, compared with MS. The number of seedlings that went on to develop green cotyledons was reduced by almost 60 % compared with MS. By contrast, 4 % glucose affected only the kinetics of development rather than the total number of seeds becoming green. A high level of glucose (6 % w/v) resulted in lower levels of greening in both wild-type and *gpt2* plants, but interestingly the impairment of greening was more severe in wild-type plants (Fig. 3). The *number* of seedlings that developed green cotyledons when grown on 6 % w/v glucose was around 25–30 % higher in plants lacking *GPT2*, despite a slower *rate* of greening.

In both wild-type and *gpt2* plants, the rate of greening was slowed with increasing glucose concentration. In both of the already delayed *gpt2* lines, the delay appeared to be slightly exacerbated by glucose. Interestingly, germination rates in *gpt2* plants were less affected by glucose concentration than wild-type. T_50_ germination and greening was calculated in seedlings grown on media containing glucose. Glucose was used at 4 % w/v in subsequent experiments, as at this concentration greening was slowed, but plants remained relatively healthy and total percentage greening was relatively unaffected (Fig. 3).

### *gpt2* plants are hyposensitive to glucose in the growth medium during germination

**Fig. 4.**
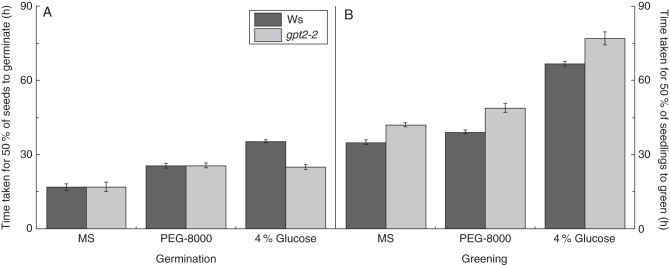
Mean time taken for 50 % (T_50_) of seeds/seedlings to achieve germination and greening on different growth media. Seeds of wild-type Ws-2 and *gpt2-2* plants were grown on half-strength MS, 4 % glucose or equivalent osmotic potential 4 % PEG-8000 half-strength MS plates. Seeds were sterilized and sown as for seedling development assays. After a 2- to 3-day stratification at 4 °C in the dark, plates were transferred to 100 μmol m^2^ s^−1^ light (8 h day/16 h night) and germination (A) and greening (B) were scored every 12 h as the emergence of the radicle and presence of green cotyledons, respectively. Percentage germination/greening over time was also graphed (see Supplementary Data Figs 3 and 4) and curves were fitted to extrapolate the average time for 50 % (T_50_) of seeds/seedlings to germinate/green. Wild-type and *gpt2* plants are as indicated in the key. Bars represent the mean of at least three replicate plates (150–300 seedlings). Error bars represent ±1 s.e.

Germination and seedling development were further examined in wild-type Ws-2 and *gpt2-2* plants grown on medium containing 4 % w/v glucose. The mean T_50_ germination and T_50_ greening were calculated to distinguish the developmental processes involved in the response to sugar (for fitted curves see Supplementary Data Figs 3 and 4). As expected, germination and greening were both slowed in wild-type plants grown on 4 % w/v glucose compared with half-strength MS (Fig. 4). There also appeared to be a cumulative response to glucose; germination occurred around 17–18 h later on 4 % w/v glucose than on MS and greening around 33 h later, suggesting that both germination and greening processes were delayed in wild-type Ws-2 plants.

The *gpt2-2* line responded differently to 4 % w/v glucose. These plants were less sensitive to glucose than wild-type in terms of germination rate, even though they had a similar greening response (i.e. *gpt2-2* seedlings greened approximately 35 h later when grown on glucose compared with MS or an osmotic control). Surprisingly, greening was delayed in *gpt2-2* plants grown on glucose, despite the hyposensitivity to sugar during germination. The mean time between germination and greening was approximately 32 h in wild-type plants and 48 h in *gpt2-2* plants, suggesting that the delay in greening seen on MS was not rescued but exacerbated by the addition of glucose to the medium.

### *gpt2-2* plants are sensitive to abscisic acid

**Fig. 5.**
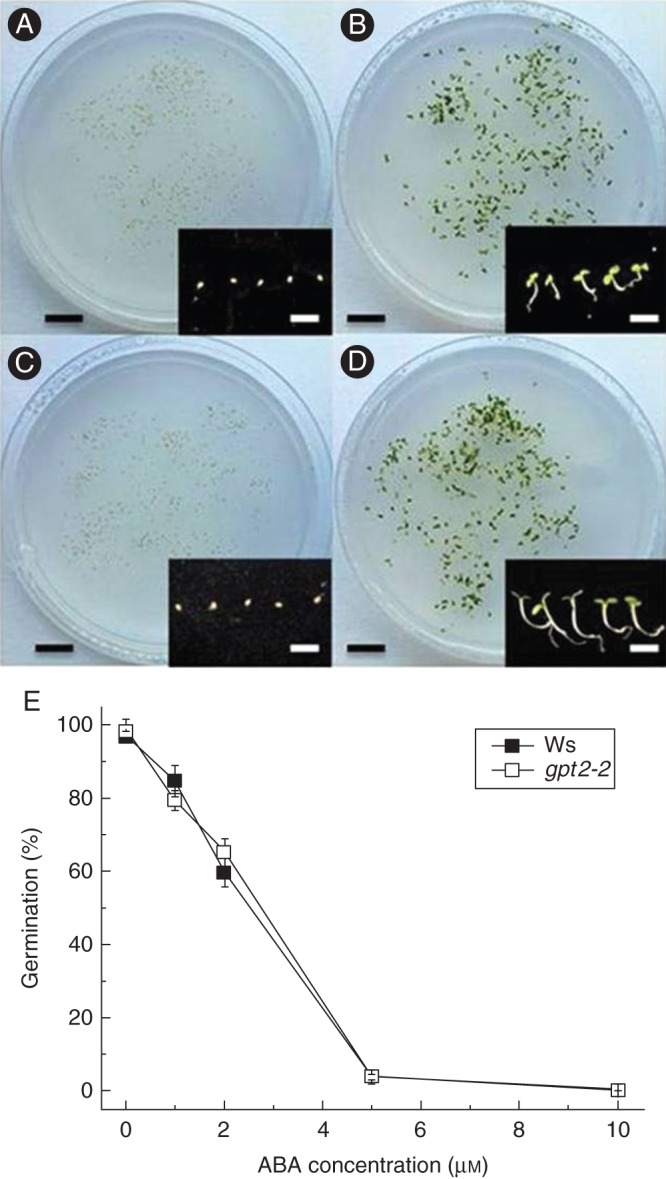
Development of wild-type and *gpt2* plants on media containing ABA. Seeds were sterilized as for seedling development assays and sown into half-strength MS or half-strength MS medium with 1–10 μm ABA. After a 2-day stratification at 4 °C in the dark, plants were transferred to 8 h day/16 h night at an irradiance of 100 μmol m^−2^ s^−1^ white fluorescent light for 7 days. Germination was scored as the emergence of the radicle (and subsequent growth). (A–D) Representative plates showing wild-type on half-strength MS (A), *gpt2* on half-strength MS (B), wild-type on half-strength MS with 10 μm ABA (C) and *gpt2* on half-strength MS with 10 μm ABA (D). Inset figures show five representative seeds/seedlings for each line/treatment. Black scale bars = 20 mm, white scale bars = 5 mm. (E) Mean percentage germination for each line/treatment on different concentrations of ABA. Each measurement represents the mean of at least three replicate plates, each plate containing at least 150 seeds. Error bars represent ±1 s.e.

A number of mutants insensitive or hyposensitive to glucose in the growth media were also affected in their response to abscisic acid (ABA), which strongly inhibits germination. To analyse the response of *gpt2* plants to ABA, seeds were sown on half-strength MS containing 1, 2, 5 or 10 μm ABA (Fig. 5). There was no difference in the response of *gpt2-2* seeds to that seen in wild-type, with germination rates of 0–1 % seen in 10 μm ABA-treated seeds, compared with rates approaching 100 % in those grown on MS alone. Intermediate concentrations of ABA had intermediate effects on seeds, with germination rates remaining relatively high on 1–2 μm ABA but severely restricted on 5 μm ABA.

### Relative expression of *GPT2* is increased during seedling development on glucose

**Fig. 6.**
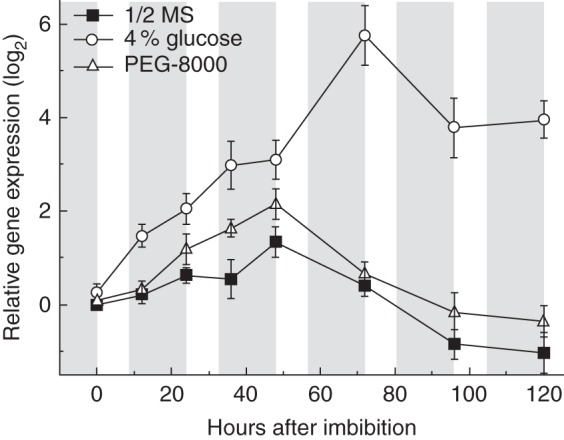
Expression of *GPT2* during early seedling development on glucose. Seeds were sterilized, stratified, sown and transferred to light as for seedling development assays, with the inclusion of 4 % glucose or PEG-8000 of the equivalent osmotic potential to the medium. *GPT2* expression was examined in seedlings after transfer to light, with samples of 200–250 seedlings flash-frozen in liquid nitrogen at the end of each photoperiod. Frozen seedlings were stored at −80 °C. Fold changes shown were calculated relative to levels of *ACT*2 expression and are displayed relative to the expression level on half-strength MS at each time point. Shaded areas represent dark periods and unshaded areas represent photoperiods. Data expressed relative to *UBC* are qualitatively identical. Error bars represent ±1 s.e. Plants grown on half-strength MS only, grown on half-strength MS with PEG-8000 with an equivalent osmotic potential to 4 % glucose, and grown on half-strength MS with 4 % glucose are as indicated in the key.

*GPT2* expression has been seen in seedlings in a number of studies and has been seen to be induced in plants grown on media containing sugars (e.g. Gonzali *et al.*, 2006). Consequently, relative *GPT2* expression was analysed using quantitative PCR during the period of greening in plants grown on glucose and the osmotic control, PEG-8000. These levels were calculated relative to expression on half-strength MS. Expression of *GPT2* in wild-type Ws-2 plants was measured, with *UBC* and *ACT2* quantified as control genes. RNA extraction was carried out on seeds and seedlings after imbibition and stratification, over a period of 120 h. *GPT2* expression was quantified relative to levels of *ACT2* expression on MS at 0 h after transfer to light (Fig. 6). Expression levels quantified relative to UBC levels were qualitatively similar (data not shown). When seeds were imbibed and stratified on MS medium, *GPT2* expression increased during germination and greening (0–48 h post-stratification), before decreasing after seedling establishment (≥72 h post-stratification). Peak *GPT2* expression coincided with the onset of greening (Figs 4 and 6). The pattern and level of expression were similar in plants treated with PEG-8000, confirming the observation that *GPT2* is not significantly induced by osmotic stress.

During seedling development on glucose, *GPT2* expression increased rapidly after stratification, showing significant increases compared with seedlings grown on MS apparent after 12 h of growth (Fig. 6). These increases continued steadily over the first 2 days post- stratification, corresponding to the period of germination in glucose-grown Ws-2 seedlings. Relative gene expression then increased rapidly between 48 and 72 h, with a 6-fold increase compared with MS controls seen at 72 h post-stratification. This peak of expression again corresponded to the period of greening in glucose-treated Ws-2 plants (60–72 h, Fig. 4).

## DISCUSSION

Successful seedling germination and establishment are crucial processes for the survival and fitness of plants. The transition from dry seed, dependent on seed reserves, to autotrophic, green cotyledons relies on a number of different regulatory mechanisms that help maximize the chances of success. Our data show that the glucose 6-phosphate translocator, GPT2, plays an important role in the early development of seedlings. Plants lacking *GPT2* are delayed in the greening of cotyledons, resulting in slower seedling growth compared with wild-type. In addition, responses to glucose are altered in *gpt2* plants; germination is accelerated, whilst greening is impeded. These phenotypes suggest that the partitioning of sugar phosphates, especially glucose 6-phosphate, between the cytosol and the plastid plays a central role in both germination and cotyledon greening.

The control of seed germination is tightly regulated to ensure that seedling development proceeds under favourable conditions. After maturation, seeds may enter a period of dormancy in which germination is delayed, despite the presence of the necessary factors for the onset of germination (Bewley, 1997). The germination of arabidopsis seeds requires a number of triggers to remove any dormancy in the seed and promote the growth of the radicle. Light, stratification and gibberellic acid production all promote germination, whilst ABA and certain sugars inhibit the process and promote dormancy (Bewley, 1997). Sugars have negative effects on seedling development at a range of concentrations. Low concentrations of sugars (0·5–2 % w/v) are known to delay germination (Dekkers *et al.*, 2004; Price *et al.*, 2003) but have a lesser effect on further development. Moderate glucose concentrations (2–6 % w/v) result in severely altered seedling growth. Seed reserves remain unmobilized, chloroplast development is slowed or halted, expansion of cotyledons is reduced and the plant may fail to develop true leaves or root systems. The reason for this response is unknown, but it is hypothesized to prevent seedling development under non-optimal conditions (Cheng *et al.*, 2002). Poor environmental conditions will result in stress and reduced development, leading to a lower respiratory rate and a lower demand for fixed carbon within the plant. This results in an accumulation of sugars within the cell, leading to a slowing of seedling development (Cheng *et al.*, 2002).

The data presented here (Fig. 4) show that *GPT2* is required for the sensitivity of germination to low to moderate concentrations of glucose in the growth medium (0·5–4 % w/v). This is consistent across both ecotypes of arabidopsis used in these experiments. High levels of *GPT2* expression have been shown to occur in response to high levels of exogenous glucose and sucrose (Gonzali *et al.*, 2006) and also to excess sucrose production within the leaf (Lloyd and Zakhleniuk, 2004). Repression of germination in response to low to moderate levels of glucose requires the expression of *GPT2* (Fig. 3). *GPT2* is expressed during seed development and is present in quiescent seeds (Fig. 6). Consequently, GPT2 protein already present in the embryo, or stored mRNA, may allow wild-type seeds to respond to exogenous glucose. The signalling pathways leading to delayed germination of arabidopsis seeds in the presence of exogenous glucose have not been fully described, but have been postulated to involve both hexokinase-dependent and hexokinase-independent mechanisms (Jang and Sheen, 1994). At low concentrations (up to 0·25 % w/v), glucose and mannose, which are both good substrates for the hexokinase enzyme, cause a delay in germination, whereas 3-*O*-methylglucose, which is a poor hexokinase substrate, does not. This suggests that a hexokinase-dependent pathway is involved in the regulatory mechanisms occurring at low concentrations. By contrast, higher concentrations of 3-*O*-methylglucose (>1·5 % w/v) have significant delaying effects on seedling development, suggesting a hexokinase-independent pathway in this case (Graham *et al.*, 1994; Jang and Sheen, 1994; Pego *et al.*, 1999). This suggests that a number of different sensing or signalling pathways are involved in the response to sugar. In this study, we see that higher concentrations of glucose (6 % w/v) inhibit germination in *gpt2* plants, whereas these are hyposensitive to lower glucose concentrations (0–4 % w/v). This is consistent with GPT2 involvement in the modulation of the hexokinase-dependent pathway.

A number of other mutants have previously been identified that are insensitive or hyposensitive to sugars during germination, including *glucose insensitive* (*gin*) and *sucrose insensitive* (*sis*) (Zhou *et al.*, 1998; Gibson *et al.*, 2001). Many of these mutants, showing altered sensitivity to sugars during development, have also been identified as being impaired in the expression of genes related to hormone regulation. Most notably, the effects of glucose on germination in arabidopsis have been tightly linked to the biosynthesis and catabolism of ABA (Chen *et al.*, 2006; Zhu *et al.*, 2011). ABA affects seedling development in the same way as high glucose concentrations, repressing germination and increasing dormancy. There seems to be considerable overlap between ABA signalling and sugar signalling pathways, with several *gin* mutants being allelic with either ABA-insensitive (*abi*) or -deficient (*aba*) mutants. *aba* mutants, deficient in ABA production and first identified by mutagenic screens for seeds able to germinate on the dormancy promoter gibberellic acid, are also insensitive to glucose (Koornneef *et al.*, 1984). However, *abi* mutants, deficient in ABA signalling, retain sensitivity to glucose with a germination response similar to that of wild-type plants (Finkelstein, 1994; Quesada *et al.*, 2000). This is consistent with the idea that the sensitivity of seed germination to glucose requires the presence of ABA but does not act via ABA signalling. Glucose levels play a part in the regulation of a number of ABA synthesis and signalling genes (Rook *et al.*, 1998; Price *et al.*, 2004), and in seedlings 140 genes were found to be similarly regulated by both glucose and ABA (Li *et al.*, 2006). Although the mechanism by which glucose acts to slow or halt germination is unknown, it has been postulated to act to increase ABA levels in seeds, either by increasing synthesis (Cheng *et al.*, 2002) or by slowing degradation (Zhu *et al.*, 2011). The sensitivity of *gpt2* seeds to ABA in the growth medium suggests that the disruption of glucose responses is not due to defects in ABA production or signalling, but to changes in the sugar-sensing pathway only. This highlights GPT2 as a transporter involved in a potentially novel sugar signalling pathway during germination in arabidopsis and suggests that GPT2 may be involved in sensing glucose levels *upstream* of ABA signalling.

During their development, seedlings have to undergo a transition from heterotropic growth, driven by stored reserves, to autotropic growth, supported by the newly formed chloroplasts. Greening of seedlings (i.e. the time from germination until the seedlings have green cotyledons) was consistently slower in plants lacking *GPT2*. This implies that the GPT2 protein plays a role in the regulation of some process in this growth phase. The peak in *gpt2* transcript levels coincided with the greening step, when green chloroplasts were being formed. This suggests that the function of GPT2 protein is in controlling the movement of sugar phosphates out of or into plastids that have already become photosynthetic, or in some way sensing the environment of these newly formed chloroplasts. Depending on the relative concentrations of sugar phosphates and free phosphate in the chloroplast and cytosol, any translocation may involve the import of free sugar phosphates into the chloroplast to be stored in starch, or, less likely, the export of sugar phosphates derived from photosynthesis, to support sucrose biosynthesis. Either way, the net effect will be to produce an environment in the cell favourable to further chloroplast development. The observation that exogenous glucose slows greening is consistent with the notion that chloroplast development is regulated in relation to available reserves in the cell. In the *gpt2* mutants, greening occurred approximately half a day later than in wild-type, implying that expression of *GPT2* buffers the reserves present, allowing chloroplast development sooner, at a higher cellular sugar concentration. In the presence of exogenous glucose, the difference between wild-type and mutant plants in greening time was similar to that seen in its absence. However, given that germination is insensitive to glucose in *gpt2* seeds, the *time from* germination to greening is substantially longer, implying that some step in this developmental stage, most likely the development of the chloroplast, is more sensitive to the presence of glucose.

In conclusion, germination of *gpt2* seeds on glucose occurred more quickly than in wild-type, but greening and cotyledon development were delayed (Fig. 4) and seedling growth was slowed (Fig. 3). *gpt2* seedlings are thus *hypo*sensitive to glucose when germinating, but *hyper*sensitive to glucose during greening. We suggest that these different functions are consistent with GPT2 playing a direct or indirect role in sugar sensing and signal transduction. Our observations provide evidence supporting the hypothesis that there are a number of sugar-signalling pathways controlling different aspects of development (Price *et al.*, 2003). During normal cotyledon development, we propose that GPT2 acts in sugar sensing or signalling by altering the partitioning of glucose 6-phosphate. Although biochemical data are not available for glucose 6-phosphate transport in seeds, the presence of *GPT2* expression and the resulting phenotypes suggest that glucose 6-phosphate transport is occurring, and therefore the partitioning of glucose 6-phosphate across the membrane will be altered. This is consistent with observed roles for GPT2 during photosynthetic acclimation and in mutants deficient in starch production (Athanasiou *et al.*, 2010; Kunz *et al.*, 2010). Only one other protein has been conclusively shown to have roles in both photosynthetic regulation and sugar sensing in seeds (Smeekens, 2000). Arabidopsis hexokinase 1 (HXK1) has been shown to act as a signal of sugar status (Jang and Sheen, 1994). HXK1 function is not required for sugar phosphorylation – other hexokinase proteins exist in arabidopsis and HXK1 knockouts have normal glucose 6-phosphate levels – but is necessary for correct responses to exogenous sugar levels (Pego *et al.*, 1999). Similarly, GPT2 is not absolutely required for glucose 6-phosphate translocation into or out of plastids (Knappe *et al.*, 2003), but is necessary for correct carbohydrate partitioning during photosynthetic light acclimation (Athanasiou *et al.*, 2010).

We propose that *GPT2* expression is also important for the correct timing of cotyledon greening and is necessary for the proper responses of seeds to exogenous glucose during the processes of germination and cotyledon development. These results both highlight the importance of sugar sensing and signalling to ensure appropriate responses to sugar levels and identify GPT2 as a transporter involved in this process. GPT2 could therefore provide a potential pathway connecting carbohydrate pools in the newly developed plastid and the cytosol. These results also add further weight to the idea that GPT2 is necessary for the correct responses to a number of changes in carbon metabolism in arabidopsis (Kunz *et al.*, 2010), either as a sensor of cellular sugar levels or in the signalling of correct responses to changes in sugar status.

## SUPPLEMENTARY DATA

Supplementary data are available online at www.aob.oxfordjournals.org and consist of the following. Figures S1: fitted curves for mean time taken for 50 % seeds/seedlings to germinate, green or become established for Ws and *gpt2-2* plants. Figure S2: fitted curves for mean time taken for 50 % seeds/seedlings to germinate, green or become established for Col 0 and *gpt2-1* plants. Figure S3: fitted curves for mean time taken for 50 % seeds/seedlings to germinate on different types of medium. Figure S4: fitted curves for mean time taken for 50 % seeds/seedlings to green on different types of medium.

Supplementary Data
