# Supplementary material for: GPT2: a glucose 6-phosphate/phosphate translocator with a novel role in the regulation of sugar signalling during seedling development
Source: Ann Bot. 2014 Jan 31;113(4):643–52. doi: 10.1093/aob/mct298 (PMC3936590; doi:10.1093/aob/mct298)
Supplement: Supplementary Data [file supp_113_4_643__index.html]

GPT2: a glucose 6-phosphate/phosphate translocator with a novel role in the regulation of sugar signalling during seedling development — Supplementary Data 

# GPT2: a glucose 6-phosphate/phosphate translocator with a novel role in the regulation of sugar signalling during seedling development

## Supplementary Data

Supplementary Data

**Files in this Data Supplement:**

- Supplementary Data - Pdf file
