# Supplementary material for: GPT2: a glucose 6-phosphate/phosphate translocator with a novel role in the regulation of sugar signalling during seedling development
Source: Ann Bot. 2014 Jan 31;113(4):643–52. doi: 10.1093/aob/mct298 (PMC3936590; doi:10.1093/aob/mct298)

SUPPLEMENTARY DATA

Fig. S1. Fitted curves for mean time taken for 50% seeds/seedlings to germinate, green or become established for Ws and gpt2-2 plants.

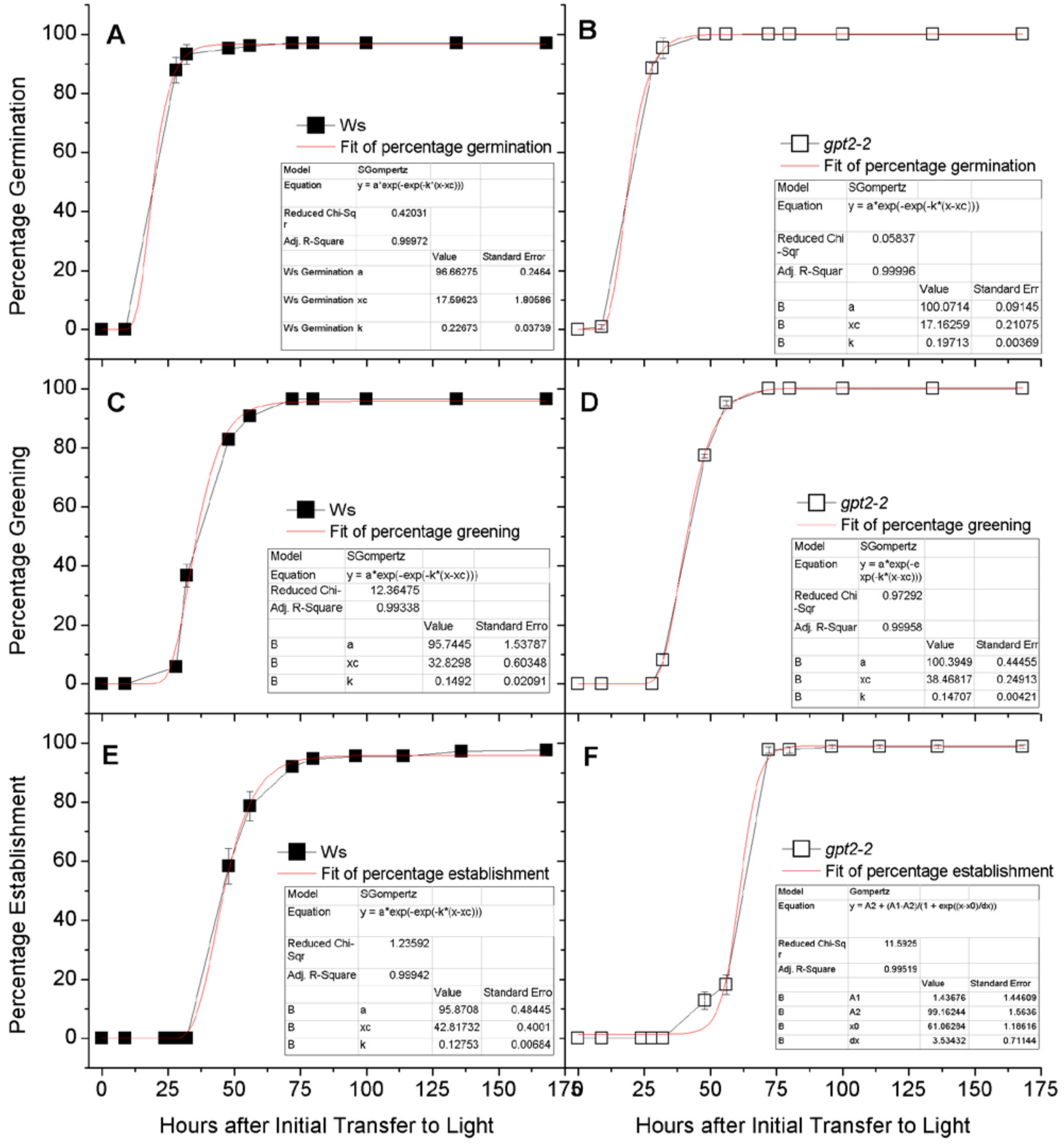

Fig. S2. Fitted curves for mean time taken for 50% seeds/seedlings to germinate, green or become established for Col 0 and gpt2-1 plants.

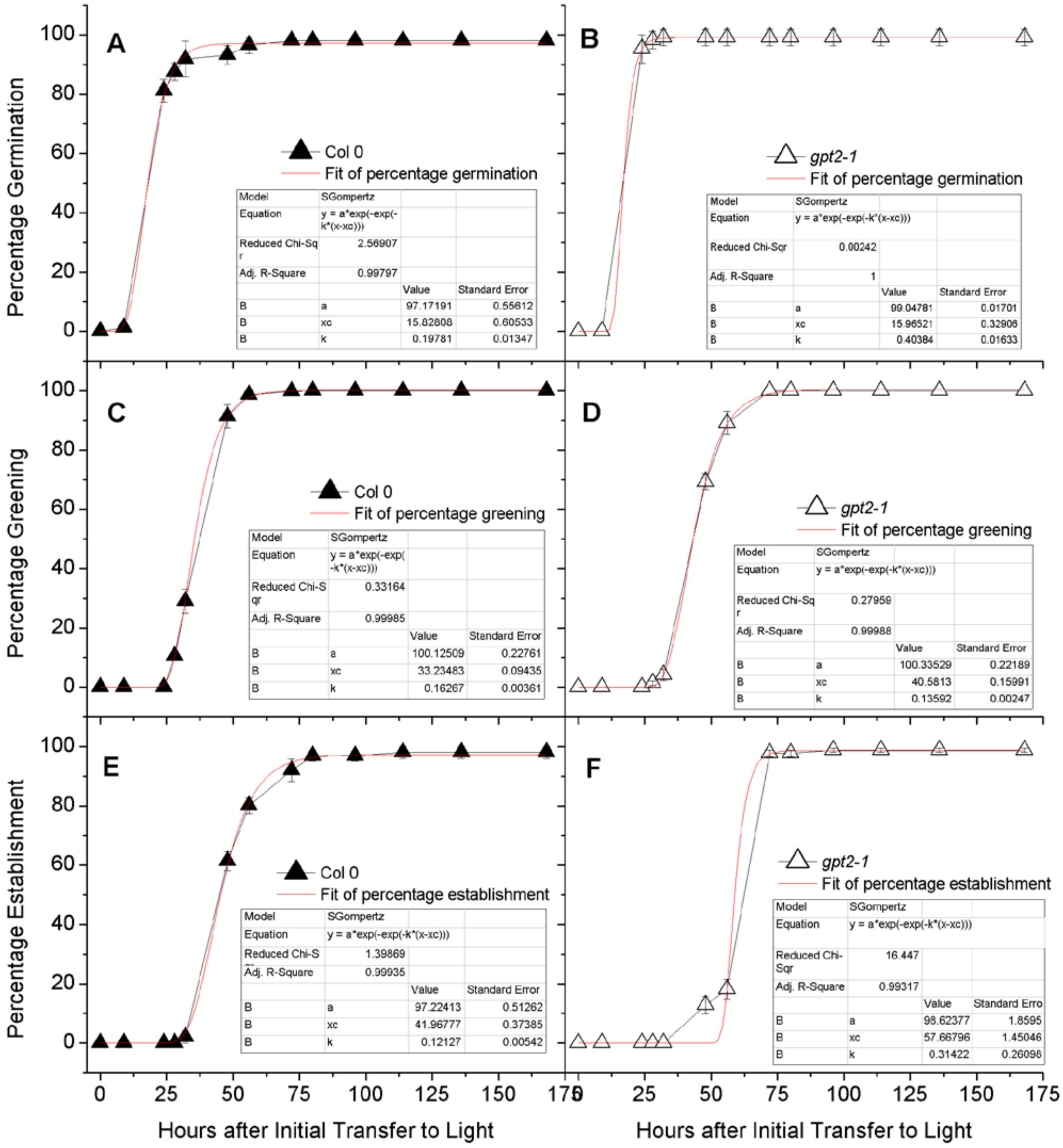

Fig. S3. Fitted curves for mean time taken for 50% seeds/seedlings to germinate on different types of media.

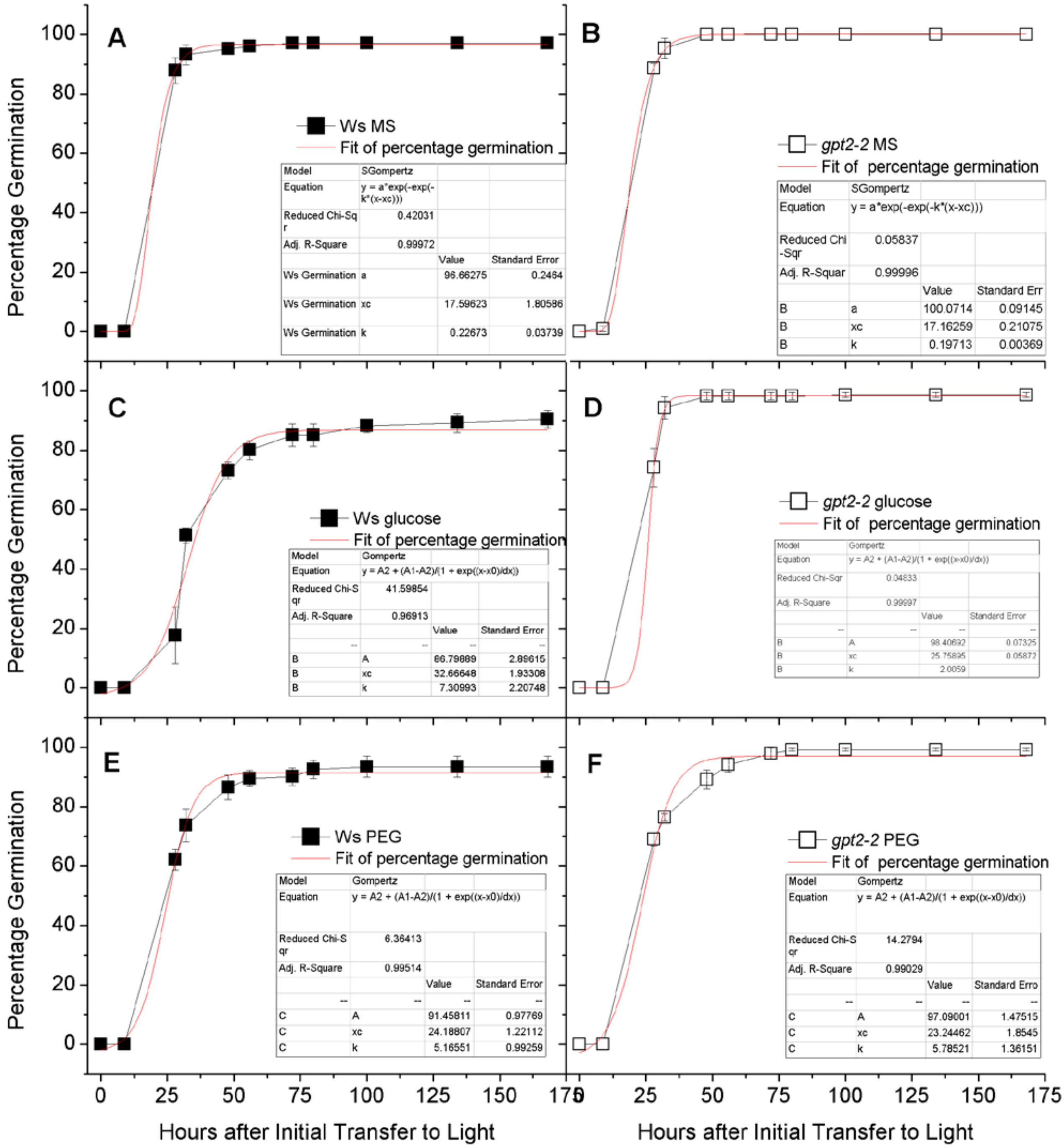

Fig. S4. Fitted curves for mean time taken for 50% seeds/seedlings to green on different types of media.

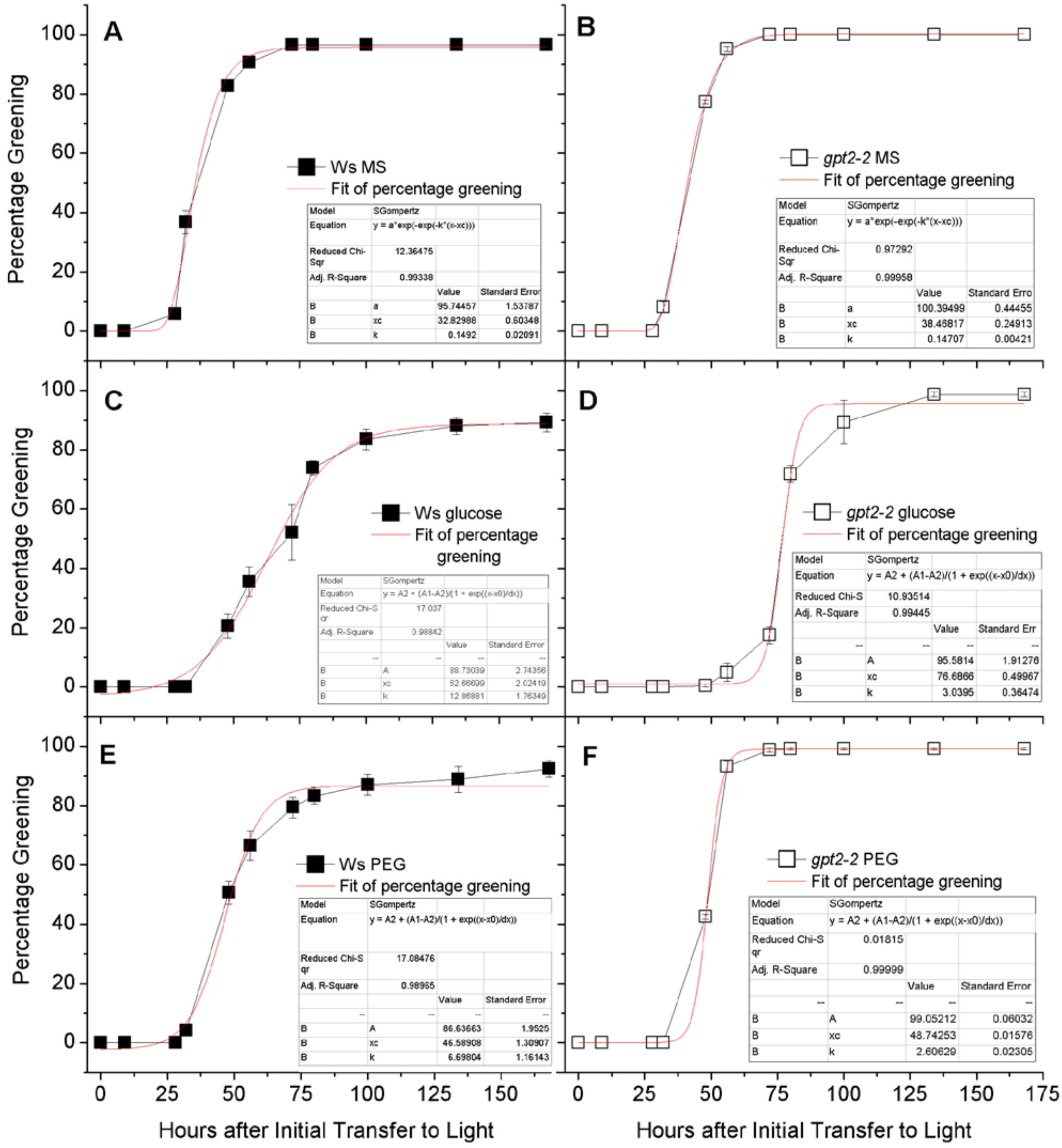

Supplement: Supplementary Data [file supp_mct298_mct298supp.pdf]
